# STEM nanoanalysis of Au/Pt/Ti-Si_3_N_4 _interfacial defects and reactions during local stress of SiGe HBTs

**DOI:** 10.1186/1556-276X-6-574

**Published:** 2011-10-31

**Authors:** Ali Alaeddine, Cécile Genevois, Laurence Chevalier, Kaouther Daoud

**Affiliations:** 1 Université de Rouen, GPM, UMR CNRS 6634, BP 12, Avenue de l'Université, 76801 Saint Etienne de Rouvray, France

**Keywords:** HBT, STEM-HAADF, EDS, EFTEM, failure, reliability

## Abstract

A new insight on the behavior of metal contact-insulating interfaces in SiGe heterojunction bipolar transistor is given by high-performance aberration-corrected scanning transmission electron microscopy (STEM) analysis tools equipped with sub-nanometric probe size. It is demonstrated that the presence of initial defects introduced during technological processes play a major role in the acceleration of degradation mechanisms of the structure during stress. A combination of energy-filtered transmission electron microscopy analysis with high angle annular dark field STEM and energy dispersive spectroscopy provides strong evidence that migration of Au-Pt from the metal contacts to Ti/Si_3_N_4 _interface is one of the precursors to species interdiffusion and reactions. High current densities and related local heating effects induce the evolution of the pure Ti initial layer into mixture layer composed of Ti, O, and N. Local contamination of Ti layers by fluorine atoms is also pointed out, as well as rupture of TiN thin barrier layer.

## Introduction

The metal contact structures are important parts of the transistors in term of device performances with the current losses and signal time delays. It needs to have optimal properties with high contact conductivity and thermo-dynamical stability to prevent contact degradation [1]. The demands for material failure analyses using high-resolution transmission electron microscopy are rapidly increasing to detect smaller defects and perform their chemical element analysis. The Ti/Pt/Au metal system continues to hold a place of choice in semiconductor electronic industry because of its high reliability level especially for discrete microwave transistors. It is well established that the titanium layer acts as an adhesive barrier layer against Pt/Au penetration and gold-silicon interaction [2]. However, Ti/Pt/Au contact failures are mainly dominated by platinum penetration into silicon. As titanium nitride (TiN) material has a lower bulk resistivity than the titanium one, it was introduced between the titanium and platinum layers for a highly stable contact [3]. Titanium nitride is quite an attractive material because it behaves as an impermeable barrier to silicon and it has high activation energy to the diffusion of other impurities [4]. Moreover, silicon nitride (Si_3_N_4_) is widely used in electronic devices for isolation between electrodes, but metal substrate cannot adhere easily to it due to its inertness. The interface diffusion and reaction of Ti/Si_3_N_4 _mainly depend on the metallization method such as deposition and thermal treatment which can enhance the adhesive force between Si_3_N_4 _and the metal layer [5]. On the other hand, the constant miniaturization of electronic components imposes to it severe service conditions such as high current densities and therefore high local temperatures. Thus, thin film interfaces are exposed to the risk of property changes that can induce physical failure mechanisms and affect the reliability of components. This work attempts to reveal, by high-performance scanning transmission electron microscopy (STEM) nanoanalysis using sub-nanometric probe size, the failure mechanisms at the Au/Pt/Ti/Si_3_N_4 _interface during local stress of SiGe heterojunction bipolar transistor (HBT). The paper is arranged as follows: "Experimental details" presents briefly the investigated devices, the coupling between high current densities induced by stress and local heating effects, and the employed experimental set-ups for microscopic failure analysis. In "Results and discussions," the structure analyses before and after stress are presented and discussed, using cross-sectional STEM observations combined with energy dispersive spectroscopy (EDS) and energy-filtered transmission electron microscopy (EFTEM). The last section draws some conclusions.

## Experimental details

### Samples and stress conditions

The SiGe HBT devices under discussion are surface-mounted components, transistors with a multi-finger structure in which a plurality of unit cells each made up of a collector, emitter, and base. These transistors, which present a low breakdown voltage BV_CE0 _= 2.3 V and DC current gain of 300, are mounted on a custom printed circuit board (PCB). To evaluate the reliability behavior within these devices and to identify the degradation mechanisms due to the electromagnetic field effects, different stress conditions have been applied. The stress procedure consists of a near-field disturbance system which includes equipment used for generation of the electromagnetic field such as a signal generator, power amplifier, and a miniature near-field probe localized above the device under test [6]. The stress has been applied on a minimum set of five devices in order to minimize the technological dispersion effects. As discussed in our previous work [7], the electromagnetic coupling phenomenon between the induced field and the micro-strip line connecting the base of the HBT is responsible for the performance degradations after stress. To understand aging impact coming from the external electromagnetic disturbance, the evolution of the current induced by coupling phenomenon in front of HBT has been studied. We found that the value of the induced current in front of the base reaches 30 mA, whereas the base breakdown current is around 200 μA [7]. In fact, temperature rise of component metal layers due to increasing current densities with associated self-heating effects can strongly affect metal connection reliability [8]. Banerjee and Mehrota [9] have studied the effect of metal self-heating on the electromigration reliability by describing the strong relationship that exists between the heat generation and the injected flow current.

To analyze the effects of the electromagnetic near-field stress on our component and in order to relate the performance degradations to the microstructural defects, the HBTs are characterized before destructive failure analysis. Among the different static and dynamic performance degradations, this paper may explain the large deviation of the dynamic performances [6,10]. In fact, the commonly associated mechanisms responsible for the dynamic characteristics degradations are the generation of a damage region at the metal interfaces (metallurgic interactions) [10,11].

### Structural analysis experiments

The characterization of the microstructures and elemental compositions of the HBT, before and after stress, were performed using a transmission electron microscope (TEM). To prepare TEM samples, the internal die of the component was firstly de-packaged by using nitric acid solution. Then, thin cross sections of specimens were prepared by focused ion beam. To avoid damage from the high-energy ion beam during sample preparation, ion beam-enhanced platinum (Pt) deposition was applied to protect the sample surface.

TEM and STEM observations were carried out on a JEOL JEM ARM 200F (JEOL Ltd., Carlsbad, CA, USA) operating at 200 kV. This microscope was equipped with a field emission gun and an aberration (Cs) corrector for the electron probe. High angle annular dark field STEM images were acquired with a camera length of 8 cm and a probe size of 0.2 nm. Elemental compositions were performed by STEM-EDS using a JEOL detector with a probe size of 0.4 nm and by energy-filtered transmission electron microscopy (EFTEM) using a Gatan GIF-Quantum spectrometer (Gatan, Inc., Pleasanton, CA, USA) with a 30-eV slit and a 9-mm GIF aperture.

## Results and discussions

**Figure 1:**
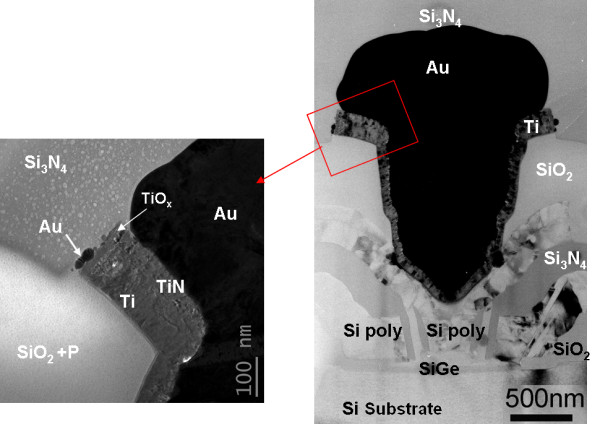
**TEM observations before stress of the emitter finger**.

We consider that a unit cell is composed of collector, emitter, and base with its intrinsic 40-nm SiGe layer under the n-doped polysilicon emitter and extrinsic layer of p-doped polysilicon under the base contact [7]. All the device fingers have been analyzed before and after stress, but the emitter results will be essentially presented in the following sections (Figure 1).

In our previous study [7], TEM observations and EDS analyses have used conventional JEOL 2000FXII equipment (JEOL Ltd.). A first approach has been then given on the structural and elemental characteristics of the device and its degradation. With the new capabilities offered by the equipment described in previous section, further investigations are proposed to better understand the failure mechanisms and their link with the original technological defects of the structure.

### Device structure and defects before stress

Our microscopic investigations are exclusively focusing on the metallic contacts and their interfaces areas because the degradations induced by our stress modes, and consequent local temperature increase, have been observed exclusively on these parts of the device.

**Figure 2:**
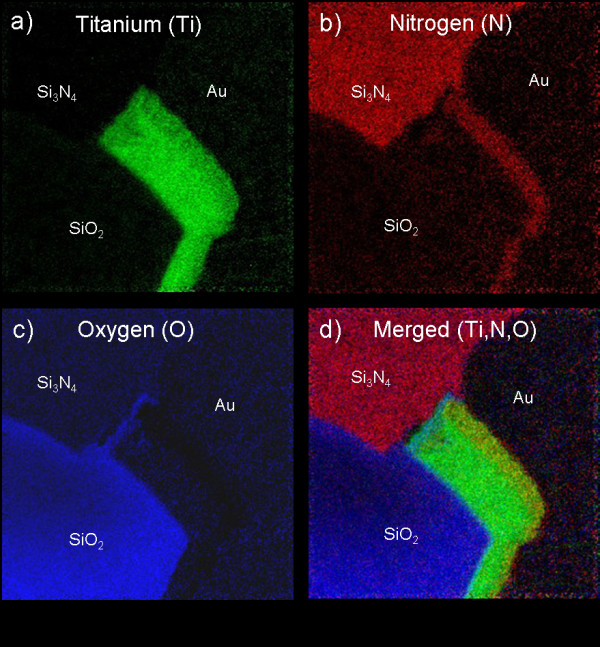
**EFTEM analysis carried out on the emitter finger**.

As indicated in Figure 1, the metallic parts are composed of pure gold (Au) in a via form, surrounded by thin titanium (Ti)-based polycrystalline films with a thickness varying between 70 and 150 nm. The insulating layers of the structure consist in SiO_2 _on the bottom with the unique Ti metal interface, and Si_3_N_4 _on the top with both Ti/TiN and Au interfaces. Pre-stress analyses indicate the presence of different layers rich in titanium (Ti) as shown in Figure 2a. It could be deduced from the preliminary observations that Ti was deposited first in the deposition sequence of metal layers and probably in two steps; its bottom interface with polysilicon gives a TiSi silicide low-resistivity ohmic contacts, while its high adhesion properties are used to ensure a better interface quality between Au metallic films and insulating layers.

Our analyses also show that a TiN diffusion barrier is present along the gold (Au) side and only with SiO_2 _interface with, in a first approximation, a thickness of 35 nm on the top side (Figure 2b). The pure titanium layer is measuring around 85 nm in thickness. In addition, the EFTEM analysis clearly reveals a thin layer of titanium oxide, of around 20 nm, at the Si_3_N_4 _interface (Figure 2c). As it will be revealed later, this interface is probably the main origin of the structural degradations.

**Figure 3:**
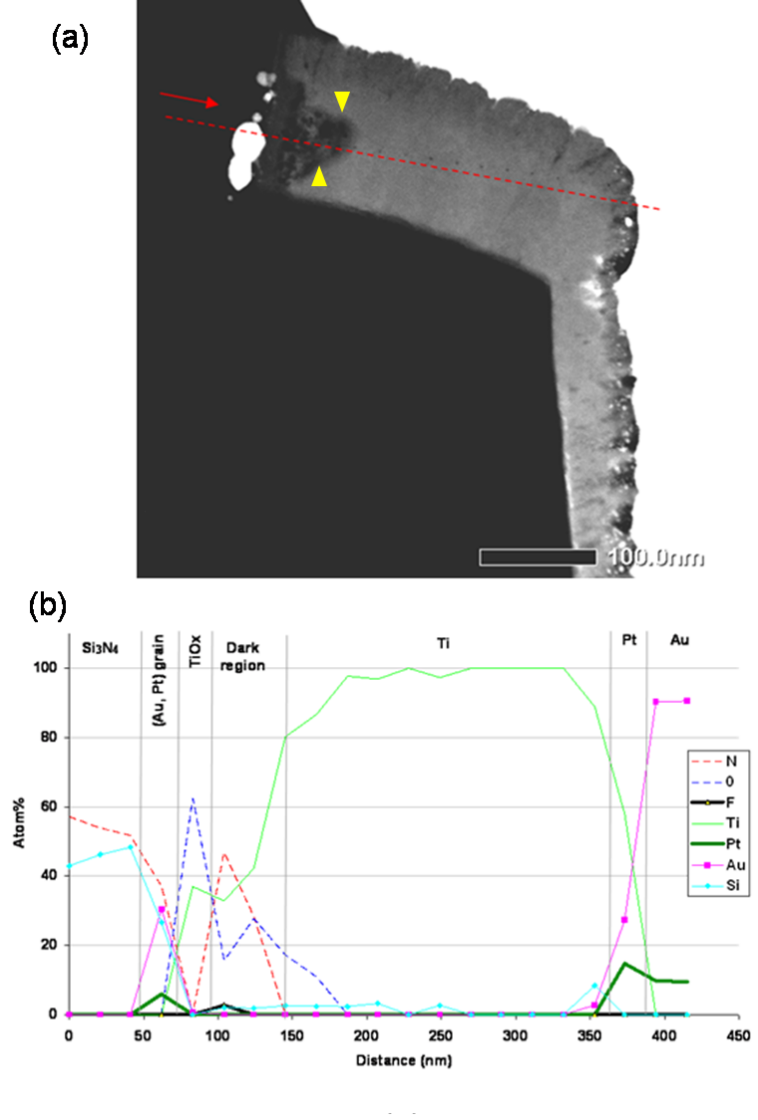
**STEM-HAADF micrograph and EDS analysis of the non-stressed emitter finger**. (**a**) The red dotted line represents the EDS line scan. (**b**) EDS line scan showing the elemental composition along the longitudinal section of the emitter contact before stress. Yellow head arrows delimit dark zone.

A STEM-HAADF image of the Au/TiN/Ti-Si_3_N_4 _interfacial area is shown in Figure 3a. The image contrast in STEM-HAADF is proportional to the atomic number *Z*. On this micrograph, a dark region, of around 50 nm, is pointed out between the titanium oxide layer and the pure titanium layer (Ti). EFTEM analysis indicates the presence of titanium in this region as shown in Figure 2a.

An EDS line scan is performed along the emitter finger with a probe size of 0.2 nm and a step of 20 nm, in order to characterize the evolution of the different element through the different layers (Figure 3a). The longitudinal section of the emitter finger is composed successively by Si_3_N_4_/(Au, Pt) grains/TiO*_x_*/dark region/Ti/Pt/Au. The dark region is a mixture containing titanium, nitrogen, and oxygen with varying concentrations and fluorine (F) in some locations. Fluorine is present only near this dark region, and its concentration of some atomic percentage (at.%) (Figure 3b).The EDS analyses highlight the presence of two peaks of thin platinum (Pt) layer simultaneously present with Au and located at the interfaces (Figure 3b).

As verified by EDS analysis of the bottom part of the contact (data not shown), the reason of Pt presence could be related to its role in the ohmic contact formation. That means that Pt should contribute to the formation of a stable TiSi phase after annealing process, as frequently reported in literature [12]. In contrast, along the present EDS analysis line, no nitrogen has been detected at the supposed TiN interface, which suggests a local rupture of TiN layer, or a very thin layer (lower than the analysis step).

In addition, EDS points show the presence of phosphorus (P) in the SiO_2 _layer, with a heterogeneous concentration distribution varying between 0 and 4 at.%. This result is consistent with the frequent use of PSG (phosphosilicate glass) process in n-doped SiO_2 _deposition [13] to improve its elasticity and increase the etch rate of the via prior to metal depositions. Combining both HAADF imaging and EDS analyses in the critical area leads to consider that the emitter metal-insulating interface is composed of Au/Pt/TiN/Ti-Si_3_N_4 _or Au/Pt/TiN/Ti-SiO_2_.

The small dark grains at the TiO*_x_*/Si_3_N_4 _interface, which are also present around the collector and the base, are composed by gold (Au) and some platinum (Pt). The thin layer of titanium oxide detected by EFTEM analysis at the Si_3_N_4 _interface could correspond to TiO_2 _composition if we observe the EDS peaks proportions in this layer (Figure 3b).

### Analyses of stressed samples

**Figure 4:**
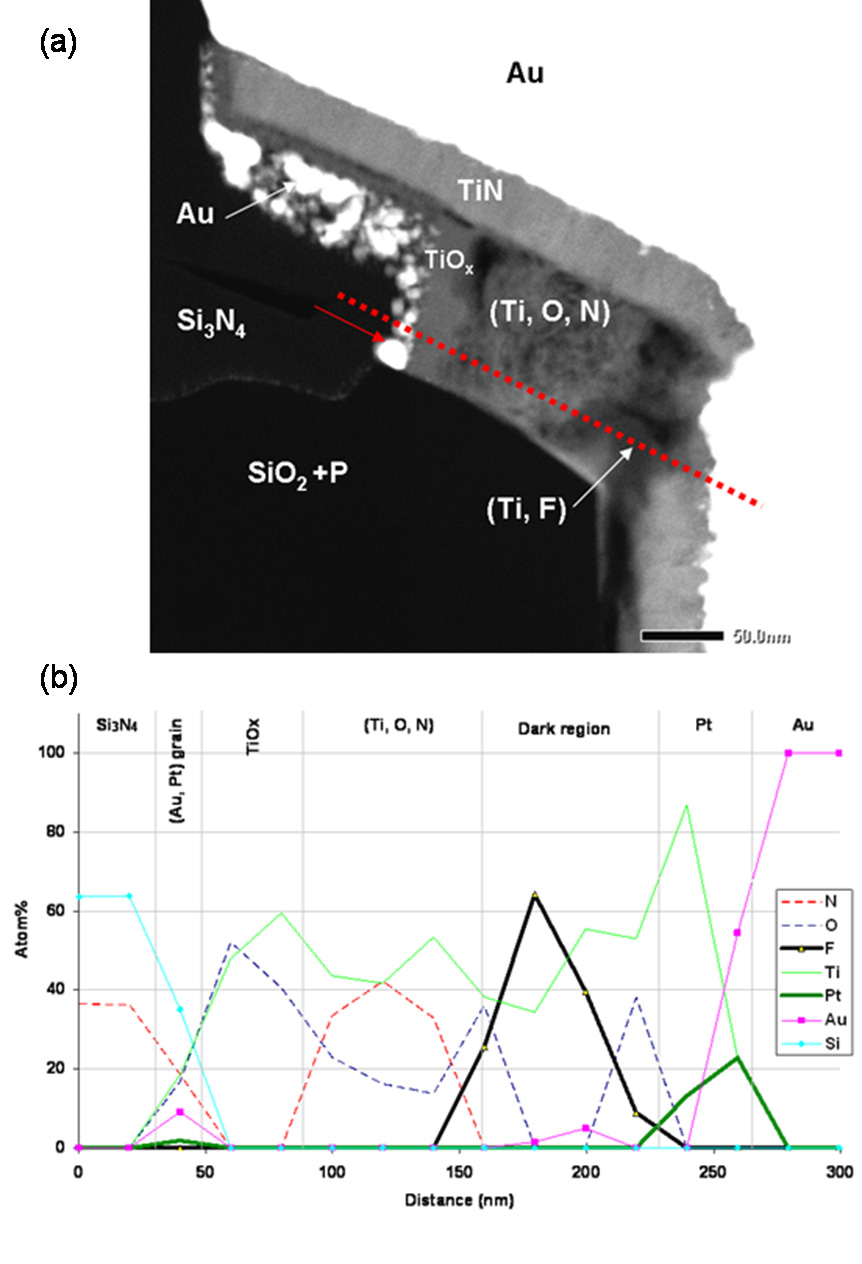
**STEM-HAADF micrograph and EDS analysis of the emitter contact stressed during 30 min**. (**a**)The red dotted line represents the EDS line scan. (**b**) EDS line scan showing the elemental evolution along the longitudinal section of the emitter contact after 30 min of stress.

In order to better understand the metallic disorders and interfacial interactions in the stressed device, STEM-HAADF observations and EDS analysis on the degraded areas were carried out. Figure 4a displays the cross-sectional image of the SiGe HBT sample selected from devices after 30 min of electromagnetic field stress.

In the micrograph, a significant evolution of the contact morphology situated around the emitter is observed. The same phenomenon is observed at the titanium layers of the base and the collector fingers and both on the right and the left sides. The main observation clearly shows a reduction of the lateral Ti layer whose length decreases from 350 to 180 nm. In this area, the initial Ti thin film has evolved into a layer composed of small grains sharply separated, but the lateral TiN layer length seems to be not affected by stress.

**Figure 5:**
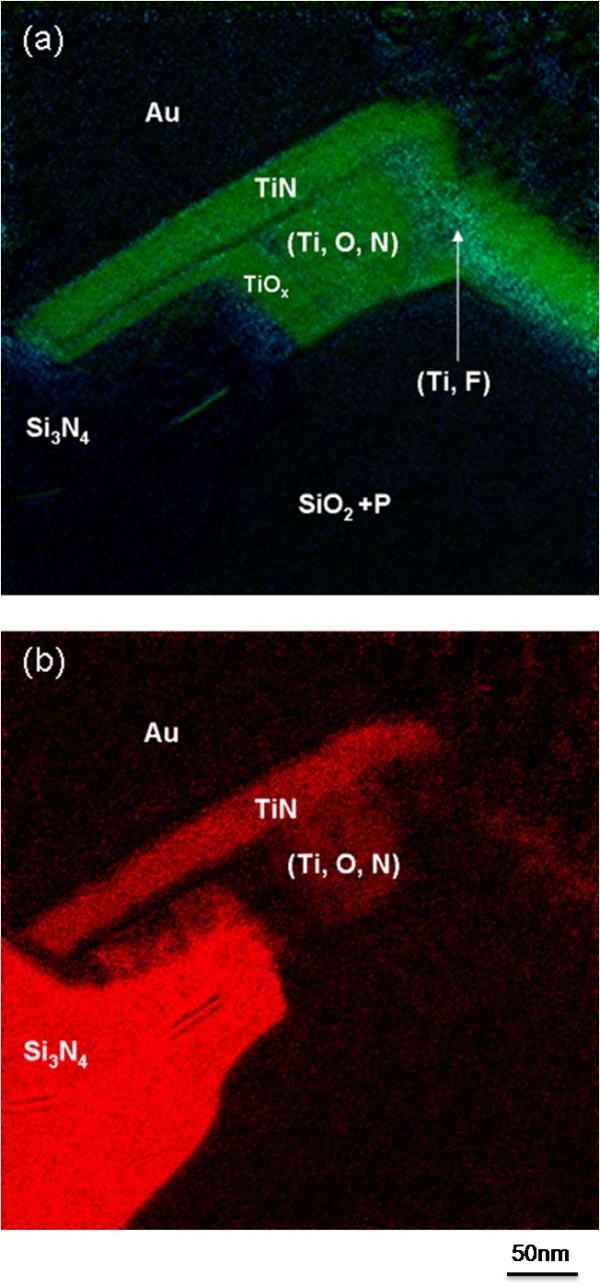
**EFTEM analysis of the emitter contact after 30 min of stress**. (**a**) Green and clear blue represent respectively titanium (Ti) and fluorine (F). (**b**) Nitrogen EFTEM analysis of the emitter contact after stress.

To determine the composition evolution of the disturbed area, EDS line scans have been carried out with a 0.2 nm probe size and a step of 20 nm (see dotted line of Figure 4a). The longitudinal section is composed, as mentioned in Figure 4b, of Si_3_N_4_/(Au, Pt) grains/TiO*_x_*/(Ti, O, N) region/dark region/Pt/Au. The pure titanium layer has completely disappeared, and instead, a very perturbed region is found. This disturbed region is composed of a (Ti, O, N) zone, which contains the same elements found before stress, and a dark region containing an important fluorine (F) concentration (Figure 5a). EFTEM analyses confirm the presence of fluorine which is detected only in the dark regions, not in the (Ti, O, N) region (Figure 5b), and its local concentration can reach more than 60 at.%.

**Figure 6:**
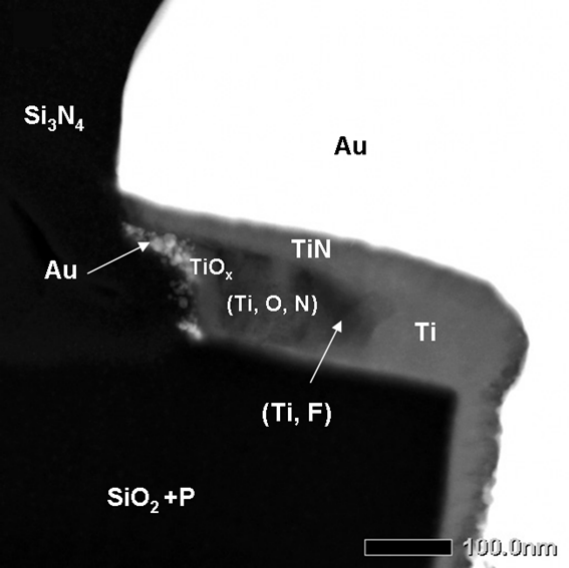
**STEM-HAADF micrograph of a base contact after a 150-min exposition under stress**.

For a comparative purpose, other samples have been analyzed and have shown an intermediate state of their interfaces evolution during stress as represented in Figure 6. On this finger where we suppose less initial interfacial defects, the pure Ti layer is partially dissolved in favor of the (Ti, O, N) and (Ti, F) regions. The fluorine peak concentration is less important, around 50 at.% max, which tends to confirm the role of the fluorine in the dissolution process of the titanium layer and the mechanisms that could depend on the initial fluorine concentration.

The titanium oxide layer (TiO*_x_*) stays present with a thickness of around 35 nm. However, this layer has migrated, like the (Ti, O, N) region, leaving a thin layer of titanium oxide (TiO*_x_*) with 10 nm in thickness and along the TiN layer (Figure 4a). A multiplication of the (Au, Pt) grains is remarkable along the Si_3_N_4_/TiO*_x _*layer interface (Figure 4a). These grains seem to be formed directly from the Au/Pt metallic part, and then, they migrate along the titanium oxide layer until reaching the Si_3_N_4_/SiO_2 _interface. Their grain size varies between 5 and 15 nm.

### Discussion of the degradation mechanisms

From previous observations, the main structural degradations seem to be: a partial or complete transformation of the initial pure Ti layer into a mixture layer, Si_3_N_4_/Ti interface deformation, AuPt migration, and TiN barrier film rupture. One of the major degradations of Ti layer is apparently related to its reaction with fluorine species. We will attempt in this comprehensive analysis to explain the origin and the behavior of this fluorine contamination. During the metallic deposition processes using fluorine gases or fluorine-based precursors, fluorine is expected to react with Ti surface to form volatile TiF*_x _*species. In fact, it has been reported by Fracassi and d'Agostino [14] that some of the produced TiF*_x _*could be left at the Ti surface after process. In addition, the authors mentioned that molecular fluorine spontaneously reacts with clean titanium and also TiO_2 _at temperatures higher than 200°C and 350°C, respectively. In addition, the presence of initial surface contamination is suspected to enhance the reaction mechanisms. Number of reported studies on the plasma etching contaminations has been described since the early 1980s [15,16]. Fluorine contamination is frequently detected by spectroscopic surface analyses techniques (ESCA, XPS...) in typical plasma etching of SiO_2 _[16]. In particular, mixture of gases containing fluorocarbons (CF_4_) and fluorohydrocarbons (CHF_3_) are commonly used for SiO_2 _etching. A great effort has been made in the past to understand the physico-chemical interactions at the solid-gas interface in reactive plasmas. Contamination may occur simultaneously by the etching process itself and/or the subsequent surface exposure to air. Herner et al. [17] has detailed in his paper the question of fluorine solubility in Ti films and the well-known "volcano" reaction in TiN/Ti, and pointed out the increase of film resistivity as one of the main effects of fluorine contaminations. In our conditions, and among the consequences of the temperature rise during stress, local increase of fluorine concentrations and its probable thermally activated reaction with Ti layer is suspected to degrade more the electrical properties of the device metal contacts. This seems to be consistent with its electrical performance degradations [7].

Au electromigration failure mechanism has been already detailed in our previous study [7]. We have shown that high current densities and resulting local heating effects during electromagnetic field stress could reach high levels at angled areas or sharp corners [18]. The importance of the electrical and thermal states of peripheral regions of the metallic layers has to be pointed out because these areas involve high local heating effects which can affect the device reliability [19]. The present additional insight aims to improve the identification of Au/Pt migration along the Ti-Si_3_N_4 _interface.

Resulting from STEM-HAADF observations, a mechanism involving the migration of Pt and Au is highlighted. The detection of both elements at the TiO*_x_*-Si_3_N_4 _interface before stress (Figure 3a) clearly shows that the metal has diffused via the interface structural defects during the deposition and/or annealing process steps. The TiO*_x _*layer may be formed by oxygen contamination before the last manufacturing process step, i.e., before the deposition step of the nitride Si_3_N_4 _between electrodes [20]. In fact, oxygen can promote the formation of the stoichiometric compounds TiO*_x _*after interaction with titanium, which can cause an undesired titanium interface [21]. Furthermore, the inertness of silicon nitride Si_3_N_4 _contributes to minimizing its adhesive force to the metal layer [5], which can affect the TiO*_x_*-Si_3_N_4 _interface quality.

Under severe stress conditions, the current densities and the temperature rise of metallic contacts, which are higher at sharp corners, enhance platinum and gold migration movement. This is confirmed by EDS analyses suggesting that the local high current density and Joule heating induce localized reactive diffusion of Au-Pt into the Ti layer to form probably Ti-Au [21] and Pt-Ti [22] intermetallic compounds. Some of these Pt/Au-Ti reactions are known to increase the resistivity of the conducting layers which directly affects the HBTs' dynamic performances. As presented in our previous work, device parameter deviations like S_11 _could be attributed in part to the rise of the metallic resistances [23].

Let us focus finally on the TiN films' reliability. It was confirmed by analyzing several samples that the lateral side of titanium nitride (TiN) layer is not affected by stress. Since the TiN compound is more stable than of pure titanium, it is commonly applied between the titanium and Au/Pt layers as a diffusion barrier in order to prevent Au-Pt migration [3,21]. In our structure, the TiN layer which is thick enough in this area appears to have successfully prevented the diffusion of Pt and Au during process and stress, at least on this lateral side. In contrast, with the absence of TiN diffusion barrier, the TiO*_x_*-Si_3_N_4 _interface defects can provide an electromigration way of Au-Pt atoms probably during thermal treatment processes. This phenomenon has been strongly enhanced by the high current density flow induced by stress [11]. The inner Ti/TiO*_x_*-Si_3_N_4 _layers have been severely degraded as shown in STEM observations. Another problematic case is the vertical TiN barrier thickness much lower than the lateral one. Our EDS profiles indicate local rupture of these very thin layers which can lead to the via interfaces degradations and species interdiffusions. This demonstrates the importance of the process used to fill the vertical interconnects in microelectronic devices [24]. Taking into account the thickness differences between lateral and vertical sides, our observations tend to predict that deposition by sputtering or physical vapor deposition has been used. Because of the induced problem of step coverage and its consequences on device reliability, deposition by chemical vapor deposition with respect of aspect ratio could be a preferred method to prevent thin film rupture.

## Conclusions

The main idea of this work is that the initial interface defects in a device are determinant parameters in the acceleration of structural degradation during stress. This has been demonstrated using high-performance STEM equipped with sub-nanometric analysis capabilities. A fine interfacial characterization combining STEM-HAADF, EFTEM, and EDS has permitted a comprehensive study of the failure mechanisms in an HBT structure submitted to local stress inducing heating effects. The results of this paper are:

1. The rupture of the TiN barrier diffusion is a crucial factor and probably the principle and first failure cause, as it could induce others.

2. The presence of fluorine contamination due to different processes (deposition, reactive etching) using this element in precursor compositions, is very problematic, and its reactivity with titanium contributes to increase the metal resistivity.

3. The strong Au and alloys electromigration along paths and interfaces without diffusion barrier could be the cause of interconnect failure (voids, shorts...).

This naturally leads to the question: how to prevent or limit these degradations? The optimization of the TiN deposition process to obtain better layer uniformity and stability seems to be a key factor. If the use of fluorine in plasma gases or chemical precursors is essential, limiting their adsorption during chemical reactions is also important. Finally, design optimization to avoid sharp angle forms and prevent local temperature increase could improve device reliability.

## Abbreviations

EDS: energy dispersive spectroscopy; EFTEM: energy-filtered transmission electron microscopy; HBT: heterojunction bipolar transistor; STEM-HAADF: scanning transmission electron microscopy-high angle annular dark field.

## Competing interests

The authors declare that they have no competing interests.

## Authors' contributions

AA drafted the manuscript and carried out part of the results interpretation. CG carried out all microscopy experiments (STEM and EDS) and contributed to the data analysis. LC participated in the samples preparation and discussion. KD took the contributions on the research guidance, discussion, and results interpretation. All authors read and approved the final manuscript.
